# Supplementary material for: Progress in Pharmacokinetics, Pharmacological Effects, and Molecular Mechanisms of Swertiamarin: A Comprehensive Review
Source: Cells. 2025 Jul 30;14(15):1173. doi: 10.3390/cells14151173 (PMC12346835; doi:10.3390/cells14151173)
Supplement: Supplementary file 1 [file cells-14-01173-s001.zip › cells-3688545-supplementary.pdf]

Abbreviation/Symbol	Full Term
**5-HT2**	5-hydroxytryptamine 2 receptor
**AA-FLS**	Adjuvant-induced arthritic rat fibroblast-like synoviocytes
**ACC1**	Acetyl-CoA carboxylase 1
**ACP**	Acid phosphatase
**AGEs**	Advanced glycation end products
**AGP**	α-1 acid glycoprotein
**Akt**	Protein kinase B
**ALP**	Alkaline phosphatase
**ALT**	Alanine aminotransferase
**AMPK**	AMP-activated protein kinase
**Ang II**	Angiotensin II
**ANIT**	α-Naphthylisothiocyanate
**AP-1 (JUN)**	Transcription factor AP-1 (c-Jun)
**APAP**	Acetaminophen
**AR**	Androgen receptor
**AST**	Aspartate aminotransferase
**AT1R**	Angiotensin II type 1 receptor
**AUC**	Area Under the Curve
**BAT**	Brown adipose tissue
**Bax**	Bcl-2-associated X protein
**Bcl-2**	B-cell lymphoma 2
**BDL**	Bile duct ligation
**Bsep**	Bile salt export pump
**BSEP**	Bile salt export pump
**C_{max}~**	Maximum plasma concentration
**CAT**	Catalase

**CDCA**	Chenodeoxycholic acid
**CFA**	Complete Freund's adjuvant
**CIRI**	Cerebral ischemia-reperfusion injury
**$CL_{z/F}$~**	Apparent terminal clearance
**cGAS-STING**	Cyclic GMP-AMP synthase-Stimulator of interferon genes
**Col-I/III**	Collagen type I/III
**COX-2**	Cyclooxygenase-2
**CS**	Cigarette smoke
**CSQ**	Combination of Swertiamarin and Quercetin
**CTGF**	Connective tissue growth factor
**DCA**	Deoxycholic acid
**DMN**	Dimethylnitrosamine
**DN**	Diabetic nephropathy
**DPN**	Diabetic peripheral neuropathy
**ECR**	Erythrocentaurin
**EGF**	Epidermal growth factor
**EMT**	Epithelial-mesenchymal transition
**eWAT**	Epididymal white adipose tissue
**ER-α**	Estrogen receptor alpha
**ERK**	Extracellular signal-regulated kinase
**ESR**	Erythrocyte sedimentation rate
**FAS**	Fatty acid synthase
**FCA**	Freund's complete adjuvant
**FRAT1**	Frequently rearranged in advanced T-cell lymphomas 1
**FXR**	Farnesoid X receptor
**GABA**	Gamma-aminobutyric acid
**GLI-1**	Glioma-associated oncogene homolog 1

**GLUT2/4**	Glucose transporter 2/4
**GPx**	Glutathione peroxidase
**GSH**	Glutathione
**GSSG**	Oxidized glutathione
**HCC**	Hepatocellular carcinoma
**HDL**	High-density lipoprotein
**HFD**	High-fat diet
**HH**	Hedgehog
**HIF-1α**	Hypoxia-inducible factor 1 alpha
**HMGR**	HMG-CoA Reductase
**HMIO**	3,4-Dihydro-5-(hydroxymethyl) isochroman-1-one
**HO-1**	Heme oxygenase-1
**HSA**	Human serum albumin
**HTPS**	Heat-transformed products of Swertiamarin
**Hyp**	Hydroxyproline
**IHH**	Indian hedgehog
**iNOS**	Inducible nitric oxide synthase
**IR-β**	Insulin receptor β-subunit
**IRS1**	Insulin receptor substrate 1
**JAK2**	Janus kinase 2
**LDL**	Low-density lipoprotein
**LLOQ**	Lower limit of quantification
**LOX**	Lysyl oxidase
**MAPK**	Mitogen-activated protein kinase
**MDA**	Malondialdehyde
**MIC**	Minimum inhibitory concentration
**MMP-3/9**	Matrix metalloproteinase-3/9

**MPO**	Myeloperoxidase
**MRP2/3/40**	Multidrug resistance-associated protein 2/3/40
**NADPH**	Nicotinamide adenine dinucleotide phosphate
**NAFLD**	Nonalcoholic fatty liver disease
**NEFA**	Non-esterified fatty acids
**NF-κB**	Nuclear factor kappa-light-chain-enhancer of activated B cells
**NLRP3**	NOD-like receptor protein 3
**NO**	Nitric oxide
**NOX4**	NADPH oxidase 4
**NQO1**	NAD(P)H quinone dehydrogenase 1
**NTCP**	Na^+-taurocholate cotransporting polypeptide
**Nrf2**	Nuclear factor erythroid 2-related factor 2
**OA**	Oleic acid
**OGDR**	Oxygen Glucose Deprivation/Re-oxygenation
**OPG**	Osteoprotegerin
**p38 MAPK**	p38 mitogen-activated protein kinase
**p-AKT**	Phosphorylated AKT
**PACP**	Prostatic acid phosphatase
**PARP1**	Poly(ADP-ribose) polymerase 1
**PBMCs**	Peripheral blood mononuclear cells
**PCNA**	Proliferating cell nuclear antigen
**PDZK1**	PDZ domain-containing protein 1
**PEPCK**	Phosphoenolpyruvate carboxykinase
**PI(3)K**	Phosphatidylinositol 3-kinase
**PI3K/Akt**	Phosphoinositide 3-kinase/Protein kinase B
**PPAR-α/γ**	Peroxisome proliferator-activated receptor α/γ
**RAGE**	Receptor for advanced glycation end products

**RANK**	Receptor activator of NF-κB
**RANKL**	Receptor activator of NF-κB ligand
**RAS**	Renin-angiotensin system
**RBC**	Red blood cell
**RIII**	Radiation-induced intestinal injury
**ROS**	Reactive oxygen species
**SHP**	Small heterodimer partner
**SMO**	Smoothened
**SOD**	Superoxide dismutase
**SREBP-1**	Sterol regulatory element-binding protein 1
**STAT3**	Signal transducer and activator of transcription 3
**SW**	Swertiamarin
$t_{1/2z}$	terminal half-fife
**TBARS**	Thiobarbituric acid reactive substances
**TC**	Total cholesterol
**TGF-β1**	Transforming growth factor-beta 1
**TH**	Tyrosine hydroxylase
**TIMP**	Tissue inhibitors of metalloproteinases
**TLR4**	Toll-like receptor 4
**TNF-α**	Tumor necrosis factor-alpha
**TRAP**	Tartrate-resistant acid phosphatase
**T-SH**	Total sulfhydryl
$t_{1/2\sim}$	Half-life
**UA**	Uric acid
$V_{z/F\sim}$	Apparent volume of distribution
**VEGF**	Vascular endothelial growth factor
**VLDL**	Very low-density lipoprotein

**WBC**	White blood cell
**XO**	Xanthine oxidase
**α-SMA**	α-Smooth muscle actin
**βFGF**	Basic fibroblast growth factor
